# Spatiotemporal Differences, Dynamic Evolution and Trend of the Coupled Coordination Relationship between Urbanization and Food Security in China

**DOI:** 10.3390/foods11162526

**Published:** 2022-08-21

**Authors:** Shan Liu, Mengyang Hou

**Affiliations:** 1 School of Management, Shijiazhuang Tiedao University, Shijiazhuang 050043, China; 2 School of Economics, Hebei University, Baoding 071000, China; 3 Research Center of Resources Utilization and Environmental Conservation, Hebei University, Baoding 071000, China

**Keywords:** urbanization, food security, regional differences, dynamic distribution, evolution trend, Dagum–Gini coefficient, kernel density estimation, spatial Markov chain

## Abstract

Scientific assessment of the coupled coordination degree between urbanization and food security (CDUFS) revealed regional differences and sources. Dynamic evolution and trends are important references for achieving a coordinated interaction between high-quality urbanization and ensuring food security. Specifically, the CDUFS was measured using prefectural panel data in China from 2000 to 2019 and the coupling coordination degree model, which revealed its spatial correlation and differentiation. On this basis, in order to examine the spatiotemporal differences and evolution of the CDUFS, the Dagum–Gini coefficient and Kernel density estimation were innovatively used to analyze its regional differences and evolution distribution. The spatial Markov chain was further employed to examine the evolution trend of the CDUFS. The study found that the CDUFS showed a downward trend in fluctuation within the low coordination interval. There was a positive spatial correlation, with a more stable distribution pattern of high–high and low–low clusters. The regional differences in the CDUFS were obvious and the overall difference has expanded. The main source of regional differences among different food functional areas was inter-regional differences, followed by intra-regional differences. The regional difference between food main producing areas and food main marketing areas was the highest. The CDUFS shows a single-peak distribution; the imbalance between regions was still prominent with a left trailing phenomenon and no convergence. The CDUFS has the stability of maintaining the original state, and the probability of leapfrogging evolution is low in the short term. Finally, the geospatial effect plays an important role in the dynamic evolution of the CDUFS.

## 1. Introduction

One of the global Sustainable Development Goals (SDGs) set by the United Nations is to achieve food security and promote sustainable agriculture [1]. Ensuring food security is also an important foundation to promote urbanization. Since the reform and opening up, China has experienced an unprecedented rapid urbanization process [2]. The rate of population urbanization increased from 17.92% in 1978 to 60.60% in 2019, with an average annual increase of 3.02%. Food security, characterized by grain yield per capita, only increased from 316.61 kg/person to 470.78 kg/person, with an average annual increase of only 0.97%, significantly lagging behind urbanization. China has the ability to guarantee the supply of grain and important agricultural products, but grain production is facing many challenges such as rising costs [3,4], resource and environmental constraints [5,6], growth in rigid demand under domestic and international market changes, climate change, etc. [7]. At present, grain production of China has stopped in 2015 at the “twelfth consecutive increase”, and there is still supply pressure [8] with a tight balance of grain supply and demand [9].

Urbanization is mainly manifested by the large-scale movement of the rural population and the demand of land expansion [10], which leads to the structural adjustment of inputs, resulting in a decrease of cropland and structural shortage of rural labor. Cropland use is also gradually non-agricultural [11]. Urbanization also leads to the growth of farm households’ non-farm income and the consumption diversification of agro-foods, resulting in the non-farming of food production [12]. Food security means maximizing the stability of food supply and ensuring that anyone has access to the food they need for survival and health, while non-agricultural and non-farming threaten food supply per capita and further affect food security. To ensure food security, adequate factor inputs and a reasonable factor allocation structure must be met, which will place a higher demand on urbanization development. If too much emphasis is placed on ensuring food security, it may cause a mobility barrier and a spatial mismatch of the urban-rural factor structure [13,14]; the development of urbanization would be unsustainable. In short, the one-sided pursuit of urbanization expansion would be a strain to food security [15]. Conversely, if food security is emphasized too much, the factor support for urbanization may be insufficient. There may be a mutually equilibrium relationship between urbanization and food security [16,17]. With the transition to high-quality socioeconomic development, a stable and coordinated state must exist between the two systems. So, it is necessary to objectively measure the coordinated relationship between urbanization and food security in China, and deeply explore its spatial-temporal differentiation, regional differences, dynamic evolution and trends. It is expected that this study can provide reference for the sustainable development of urbanization and food security in China and other developing countries.

Existing studies have launched rich exploration of the relationship between urbanization and food security, but mainly focused on the unidirectional impact of urbanization [17]. The unbalanced development of urbanization has created real problems and practical contradictions in food security [11], but urbanization from different perspectives have different impacts on food security. Xu found that economic urbanization and population urbanization in Jiangsu province can promote food security, while land urbanization and consumption urbanization have different degrees of negative effects on food security [18]. While a study at the national level found that urbanization has a significant positive impact on food security, the impact varies across food functional areas, with urbanization in the main food marketing areas having a significant negative impact on food security [19]. In terms of the two-way interaction between urbanization and food security, Yao et al. quantitatively analyzed the relationship between the urbanization system and food security system, the relationship between the two systems is increasing [20]. Zhu et al. measured the coupling coordination between new urbanization and food security in Henan province and found that the coordination degree of the two systems in Henan province basically achieved a leap of “near disorder → moderate coordination” [21]. Hou et al. believed that the coupling coordination between urbanization and grain production in China has just entered a highly coordinated range, and there is still much room for improvement [22]. Meanwhile, the study of the relationship between the two systems involves the interaction between urbanization and cultivate land pressure, total factor productivity [23,24,25], coordinated development between agricultural modernization and urbanization [26,27].

In general, the existing studies provide a solid theoretical foundation for an exploration of the relationship between these two systems. However, it is still insufficient to excavate the change laws, so there is still room for exploration. First, regarding research scale, most of the literature focuses on analysis at the provincial level [20] or in a particular province, which may not accurately reflect the imbalance degree of urbanization and food security within province. Second, regarding research content, most of the literature analyzed the one-way impact of urbanization on food security but there is still a lack of systematic study on the coordination between the two. Third, regarding research depth, the studies on spatial-temporal differences mostly use GIS technology to compare and analyze the different spatial distribution of time-series variability [21,22]. The examination of regional differences and evolution trend is still insufficient.

In total, this study examines in more detail on a smaller dimension at the prefecture level [28] to overcome the homogenization error due to provincial macro data. Specifically, using the panel data of 330 prefecture-level and above cities in China from 2000–2019 as the research sample. Based on measurement of the comprehensive level of urbanization by the entropy method, the coordination degree between urbanization and food security (CDUFS) is measured by the coupled coordination model and reveals its spatial-temporal differentiation through the exploratory spatial data analysis (ESDA). From the perspective of different food functional areas, the regional differences and distribution dynamics of the CDUFS are analyzed by the Dagum–Gini coefficient and Kernel density estimation, and then the spatial Markov chain method is used to analyze its evolution trend. The study purpose was to grasp the evolution law of the interaction between urbanization and food security in China at the macro level. This is an important theoretical reference for other developing countries facing food security problems in the process of urbanization and for achieving a balance relationship between economic development and food security.

## 2. Materials and Methods

### 2.1. Variable Definition

In order to scientifically assess the coordinating relationship between urbanization and food security, a clear definition and measurement of both is needed. Specifically:

(1) Urbanization. Urbanization is a comprehensive system with multi-dimensional features such as population mobility, urban land growth and non-agricultural economy development [20]. A single-dimensional population urbanization is different to reflect the complex characteristics of urbanization [29,30]. With reference to existing studies and data acquisition at the prefecture-level, indicators are selected from population, land, and economy to assess the comprehensive level of urbanization [31,32]. Population urbanization is characterized by the urbanization rate of resident population. Land urbanization is equal to the proportion of the urban built-up area to land area. Economy urbanization is equal to the proportion of non-agricultural industries to GDP. The entropy method is used to assign weights to indicators and conduct comprehensive evaluation to avoid the subjectivity of artificial assignment [33]. Limited to space, the detailed calculation can be found in the cited literature. The relevant indicators are standardized by the polarization method before calculation [34].
(2) Food security. Food security is also a comprehensive system containing multi-dimensional indicators such as self-sufficiency rate, reserve level, and per capita food possession [35,36]. However, some indicator data are difficult to obtain at the prefecture level, so from the perspective of per capita output scale, we use food output per capita (food production/total population) to portray food security. The reason is that the fundamental strategy to ensure food security remains to increase the output capacity per capita of food to stabilize supply. In addition, the international food security standard line is also measured by per capita food possession [37].

### 2.2. Data Sources

The study sample in this paper is panel data of 330 prefecture-level and above cities from 2000–2019. Tibet, Hong Kong, Macao and Taiwan are not involved due to the limitation of data availability. The data involved are mainly obtained from the China Urban Statistical Yearbook, China Regional Economic Statistical Yearbook and provincial statistical yearbooks (https://data.cnki.net/Yearbook, accessed on 20 May 2022). The missing data are supplemented by prefecture—level municipal statistical yearbooks and statistical bulletins. The land use data is obtained from the European Space Agency Climate Change Initiative (CCI) global land cover product data (www.esa-landcover-cci.org, accessed on 20 May 2022) with spatial-temporal resolutions of annual scale and 300 m × 300 m [38]. Table 1 shows the brief descriptive statistics.

**Figure 1:**
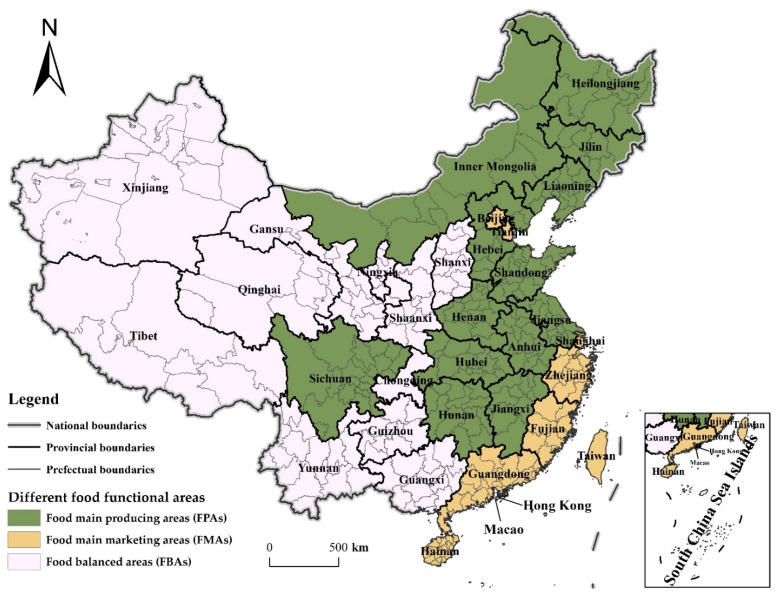
The distribution of different food functional areas. Note: This figure is drawn by the authors themselves based on ArcGIS.

In addition, to examine the regional differences of the CDUFS, the provinces where the prefecture-level cities are located nationwide are divided into three different food functional areas (Figure 1), food main producing areas (FPAs), food main marketing areas (FMAs) and food balanced areas (FBAs), according to different agricultural production endowments and subjects [22].

### 2.3. Research Methods

#### 2.3.1. CDUFS Measurement: Coupling Coordination Degree Model

The coordination degree between urbanization and food security (CDUFS) is measured by the coupling coordination degree model. The equation is as follows.

(1) $$C=2*{\left\{\frac{U_{1}\times U_{2}}{{\left(U_{i}+U_{j}\right)}^{2}}\right\}}^{1/2}$$

(2) $$\left\{ \begin{array}{l} D=\sqrt{C\times T}\\ T=aU_{1}+bU_{2} \end{array}\right.$$

In Equations (1) and (2), *C* is the coupling degree, and *C* ∈ [0,1]; *U*_1_, *U*_2_ are urbanization and food security, respectively. *D* is the CDUFS, *T* is the comprehensive coordination index between urbanization and food security, reflecting the synergistic effect of the two, and *T* ∈ (0, 1) to ensure *D* ∈ (0, 1). *A*, *b* are the pending coefficients, and *a* + *b* = 1.

Treating urbanization and food security as equally important, set *a* = 0.5, *b* = 0.5. *D* is classified into four coordination types of Low (0 ≤ *D* < 0.3), Moderate (0.3 ≤ *D* < 0.5), High (0.5 ≤ *D* < 0.8), and Extreme (0.8 ≤ *D* < 1) [39].

#### 2.3.2. Spatiotemporal Characteristics: Exploratory Spatial Data Analysis (ESDA)

Exploratory Spatial Data Analysis (ESDA) is a traditional method used to test spatiotemporal characteristics through spatial autocorrelation. Spatial autocorrelation is to explore spatial clustering or spatial anomalies by describing and visualizing the spatial pattern of a thing or phenomenon, which usually includes global spatial autocorrelation and local spatial autocorrelation. Global spatial autocorrelation is mainly used to analyze the spatial correlation of an element as a whole, which usually measured by Global Moran’s *I* [40]. Its calculation equation is:

(3) $$I=n\sum_{i=1}^{n}\sum_{j=1}^{n}w_{ij}(x_{i}-\bar{x})(x_{j}-\bar{x})/\sum_{i=1}^{n}\left(x_{i}-\bar{x}\right)\sum_{i=1}^{n}\sum_{j=1}^{n}w_{ij}$$

In Equation (3), *n* is the number of the study area; *x* is the CDUFS; $\bar{x}$ is its mean value; *w_ij_* is the spatial weight matrix constructed from the reciprocal of geographical distance and normalized by rows, $\underset{j}{\sum }w_{ij}$ = 1. *I* ∈ [–1,1], *I* > 0, there is a spatial positive correlation in CDUFS; *I* < 0, there is a spatial negative correlation in CDUFS; *I* = 0, CDUFS is randomly distributed in space.

Local spatial autocorrelation reflects the cluster or outlier of attribute values between a region and its neighbors [41], and is used to observe the spatial imbalance, which is usually measured by Local Moran’s *I*. The specific measure is LISA (Local Indicators of Spatial Association). Its calculation equation is:

(4) $$I_{i}=z_{i}\sum_{j}w_{ij}z_{j}$$

In Equation (4), *z_i_*, *z_j_* are the variance normalized values of CDUFS of city *i*, *j*, respectively.

LISA can classify CDUFS into four different types: High-High Cluster (H-H), where the CDUFS between a region itself and its neighboring regions are both high; Low-Low Cluster (L-L), where the CDUFS between a region itself and its neighboring regions are both low; High-Low Outlier, where the CDUFS in a region itself is high but its neighboring regions is low (H-L); Low-High Outlier (L-H), where the CDFUS in a region itself is low but its neighboring regions are high.

#### 2.3.3. Regional Differences: Dagum–Gini Coefficient

The Dagum–Gini coefficient can analyze regional differences and decomposition of the CDUFS [42]. It can fully consider the distribution of sub-samples and decompose the overall Gini coefficient into intra-regional variation contribution, inter-regional variation contribution and transvariation density contribution [43], which can overcome the limitations of the traditional Gini coefficient and Theil index, effectively solving the issue of cross-overlap of sample data and the source of regional differences [44]. Before the calculation, the study object needs to be divided into different regions. The Dagum–Gini coefficient (G) for all regions is calculated as:

(5) $$G=\sum_{j=1}^{k}\sum_{h=1}^{k}\sum_{i=1}^{n_{j}}\sum_{r=1}^{n_{h}}\left|y_{ji}-y_{hr}\right|/2n^{2}\bar{y}$$

In Equation (5), *yji* (*y_hr_*) is the CDUFS of any region within *j* (*h*), $\bar{y}$ is the average of the CDUFS, *n* is the number of cities, *k* is the number of regions, *j* and *h* are region subscripts, *i* and *r* are the city subscripts.

In decomposing *G* by subgroups, first, the regions are ordered according to the mean value of the CDUFS, that is, $\bar{y_{h}}\leq \bar{y_{j}}\leq \ldots \leq \bar{y_{k}}$, then, the *G* is decomposed into three components: intra-regional variation contribution (*G_w_*), inter-regional variation contribution (*G_nb_*) and transvariation density contribution (*G_i_*), which meet *G* = *G_w_*+ *G_nb_*+ *G_i_* [45]. The *G* portrays the size and source of the relative difference in the CDUFS.

(6) $$G_{w}=\sum_{j=1}^{k}G_{jj}p_{j}s_{j},\;G_{nb}=\sum_{j=2}^{k}\sum_{h=1}^{j-1}G_{jh}(p_{j}s_{h}+p_{h}s_{j})D_{jh},\;G_{t}=\sum_{j=2}^{k}\sum_{h=1}^{j-1}G_{jh}(p_{j}s_{h}+p_{h}s_{j})(1-D_{jh}),\;G_{jj}=\frac{\frac{1}{2{\bar{y}}_{j}}\sum_{i=1}^{n_{j}}\sum_{r=1}^{n_{j}}\left|y_{ji}-y_{hr}\right|}{n_{j}^{2}},\;G_{jh}=\sum_{i=1}^{n_{j}}\sum_{r=1}^{n_{h}}\frac{\left|y_{ji}-y_{hr}\right|}{n_{j}n_{h}({\bar{y}}_{j}+{\bar{y}}_{h})},\;D_{jh}=\frac{d_{jh}-p_{jh}}{d_{jh}+p_{jh}},\;d_{jh}=\int_{0}^{\infty }dF_{j}(y)\int_{0}^{y}\left(y-x)dF_{h}(x\right),\;p_{jh}=\int_{0}^{\infty }dF_{h}(y)\int_{0}^{y}\left(y-x)dF_{j}(x\right)$$

In Equation (6), *p_j_* = *n_j_*/*n*, *s_j_* = *n_j_*$\bar{y_{j}}$*/n*$\bar{y}$, *D_jh_* is the relative impact of the CDUFS in regions *j* and *h. d_jh_* is the difference in the variation of the CDUFS among regions, which represents the mathematical expectation of all sample values sum of *y_ji_* − *y_hr_* > 0 in regions *j* and *h*. *p_jh_* is the hypervariable first-order moment, which represents the mathematical expectation of all sample values sum of *y_hr_* − *y_ji_* > 0 in regions *j* and *h*. *F_j_* (*F_h_*) is the cumulative density distribution function of region *j* (*h*).

#### 2.3.4. Dynamic Distribution: Kernel Density Estimation (KDE)

Kernel density estimation (KDE) is a nonparametric method that uses continuous density curves to describe the distribution of random variables. It can be used to analyze the distribution dynamics, polarization trends and distribution extension of CDUFS [46]. Suppose the density function of random variable *x* is *f(x)*, for *x* with *n* independent and identically distributed observations, *x_1_*, *x_2_*, …, *x_n_*, and $\bar{x}$ as its mean, the estimate of the Kernel density function is

(7) $$f(x)=\frac{1}{nh}\sum_{i=1}^{n}K(\frac{x_{i}-\bar{x}}{h})$$

In Equation (7), *n* is the study samples, *h* is the bandwidth, and *K* (·) is the stochastic kernel function, which is a weighting function or a smoothing transformation function, and the Gaussian (Normal) kernel function is used in this paper. The choice of bandwidth determines the smoothing degree of estimated density function. The optimal bandwidth must be chosen in a trade-off between the variance and bias of kernel estimation so that the mean square error is minimized.

#### 2.3.5. Evolution Trend: Spatial Markov Chain

The Markov chain is a special kind of stochastic process, which can measure the state of an event occurrence and its development trend by constructing a state transfer probability matrix. The Markov chain follows the principle of “no aftereffect”, and the conditional distribution of state *X_t_* depends only on state *X_t−1_* [47]. The evolution process of many economic phenomena such as the CDUFS possess “no aftereffect”. We suppose that *P_ij_* is the transition probability of the CDUFS from state *i* in year *t* to state *j* in year *t* + 1, and it can be estimated by transition frequency approximation, i.e., *P_ij_ = n_ij_/n_i_*. Where *n_ij_* refers to the number of cities that transferred from state *i* to state *j*, and satisfy $\underset{j}{\sum }P_{ij}=\underset{j}{\sum }P\left\{X_{n+1}{=j|X}_{n}=i\right\}$ = 1. If the CDUFS is divided into *N* types, the state transition probability matrix can be constructed as *N* × *N*. Moreover, the transition direction (downward, unchanged, upward) is defined according to the change of the CDUFS types.

The spatial Markov chain introduces spatial lag into the transition probability matrix, which makes up for the neglect of the spatial correlation effect in the traditional Markov chain and can be used to reveal the intrinsic connection between spatial-temporal evolution of the CDUFS and its spatial context. Taking the spatial lag type of a region in the initial year as the conditional basis, the spatial Markov chain can decompose the traditional *N* × *N* transfer probability matrix into *N* × *N* × *N* transition probability matrix, so as to analyze the spatial dynamic evolution trend of the CDUFS and the impact of spatial effect under different geographical background conditions [48].

## 3. Results

### 3.1. Spatiotemporal Correlation and Differentiation

#### 3.1.1. Global Spatial Autocorrelation

**Figure 2:**
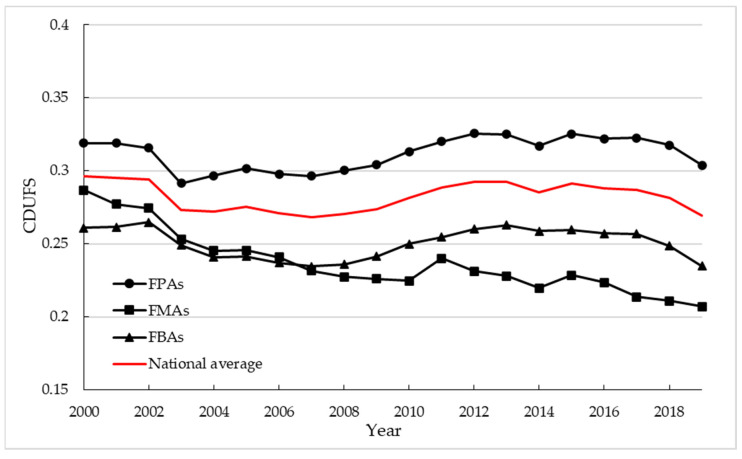
Time variation of CDUFS in different food functional areas. Note: This figure is drawn by the authors themselves based on the calculation results.

After measuring the CDUFS at the prefecture-level city, the national average CDUFS changed overall from 0.2965 to 0.2691, showing a significant downward trend and always fluctuating within the low coordination interval. The CDUFS showed an obvious regional differentiation in different food producing areas (Figure 2), mainly in the following ways: FPAs > FMAs > FBAs. Specifically, FPAs belonged to moderate coordination, while the FMAs and FBAs belonged to low coordination, and both showed a fluctuating downward trend, with the FMAs showing a more prominent downward trend.

Related studies have confirmed that the spatial mobility of factors makes a significant spatial correlation to both urbanization and food production [49,50]. Based on the distance relationship between regions, Moran’s I for the CDUFS from 2000–2019 was calculated. The Moran’s I ∈ [0.353, 0.674] of the CDUFS at the prefecture-level city all passed the significance test at the 1% level (Table 2). Moran’s I basically showed a steady increasing trend. The CDUFS had a significant positive spatial correlation and dependence, and this positive correlation in space showed a stable and continuous strengthening.

#### 3.1.2. Local Spatial Autocorrelation

**Figure 3:**
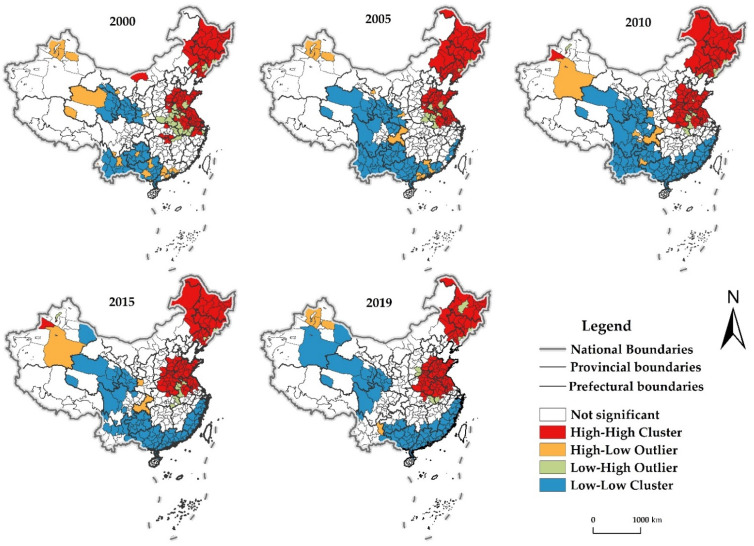
LISA distribution of CDUFS. Note: This figure is drawn by the authors themselves using ArcGIS based on the calculation results.

To further portray the spatial differentiation of the CDUFS, the years 2000, 2005, 2010, 2015 and 2019 were selected for LISA analysis (Figure 3). The FPAs in the Northeast Plain and the Huang-huaihai Plain showed H-H significantly. The FBAs or FMAs in the Northwest, the Southwest and the South China coast showed significant L-L, while showing H-L and L-H sporadic distribution around H-H and L-L, but fluctuations also occurred in local areas.

(1) High-High Cluster (H-H). Areas with H-H are mainly distributed in the FPAs such as the Northeast Plain, the North China Plain, and the middle and lower reaches of the Yangtze River. The coverage of H-H in general also showed a steady expansion of changes. The H-H areas in 2000 were mainly located in the FPAs of Northeast Plain, North China Plain and the lower Yangtze River. By 2019, the distribution expanded significantly to the FPAs in the center region, especially Hubei and Henan. Overall, the distribution of H-H was more stable, and its distribution range was gradually expanding to the FPAs in the middle reaches of the Yangtze River.
(2) Low-Low Cluster (L-L). Areas with L-L were steadily at the Northwest, Southwest and Southeast coast, concentrated in the FBAs and FMAs. In terms of changes in their coverage, it showed a stable expansion in the Northwest, expansion followed by contraction in the Southwest, and obvious expansion in the Southeast coastal. In general, the distribution pattern of H-H was relatively stable, but the southwest region shrunk significantly, and the distribution range gradually expanded to the FMAs of the Southeast coast.
(3) High-Low Outlier and Low-High Outlier (H-L, L-H). Areas with H-L and L-H of CDUFS were less distributed and sporadic, concentrated in the peripheral neighboring areas of H-H and L-L. The interaction between areas weakened the CDUFS to a certain extent. Individual areas in Qinghai have changed from H-L to L-L, which means that its urbanization and food security are faced with great challenges. Other areas with H-L are mainly at Xinjiang, Yunnan, Guangxi, etc., concentrated in the periphery of L-L. The areas with H-L in Yunnan and Guangxi are gradually disappearing while areas with L-H include the Daxinganling in Heilongjiang, mainly at the periphery of H-H.

### 3.2. Regional Differences and Decomposition

Although the analysis of spatiotemporal correlation and differentiation can visually portray the heterogeneous distribution of the CDUFS among regions, it is difficult to further reflect the extent of regional differences and their causes. Therefore, the Dagum–Gini coefficient method was used to systematically analyze the overall differences in the CDUFS, the differences in different food functional areas and their sources.

#### 3.2.1. Overall Regional Differences

**Figure 4:**
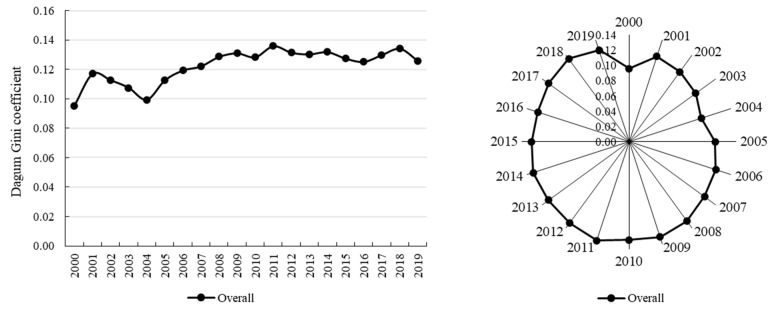
Overall regional differences in the CDUFS. Note: This figure is drawn by the authors themselves based on the calculation results.

The Dagum–Gini coefficient of CDUFS ranged from 0.0953 to 0.1360, with a mean value of 0.1223 during study period, implying that the coordination relationship between urbanization and food security exhibited a certain spatial disequilibrium. After a brief oscillation until 2004, the Gini coefficient of the the CDUFS showed an upward trend and reached a maximum in 2011 (0.1360), after which it declined slightly in fluctuations (Figure 4). The Gini coefficient of the CDUFS increased by 0.0305, or 32.00%, with a more significant increase, so the overall regional differences of the CDUFS in China have expanded.

#### 3.2.2. Regional Differences and Their Decomposition

For intra-regional differences (Table 3), the mean values of the Gini coefficient of the CDUFS in the FPAs, FMAs and FBAs were 0.0933, 0.0958 and 0.1181, respectively, and the internal differences of coupling coordination in FMAs were higher than those in the FPAs and FBAs. The intra-regional differences of the CDUFS in the three food functional areas had different trends, but compared with 2000, they all showed an overall increase, indicating that the internal differences of different food functional areas of CDUFS have expanded. The Gini coefficient of FPAs changed from 0.0742 in 2000 with an increasing and then decreasing trend to 0.0774 in 2004, and then gradually increased steadily to 0.0882 in 2019, with an overall increase of 18.87%. The Gini coefficient of FMAs, except for the anomaly in 2011 (0.1433), basically showed a steady increase, from 0.0750 to 0.1126 year-on-year, an overall increase of 50.13%. The Gini coefficient of FBAs changed from 0.0959 in 2000 to 0.0912 in 2004 first increasing and then decreasing, then increased to 0.1003 in 2019, with a relatively small increase of 4.59% overall. In addition, the expansion trend of internal differences of the CDUFS was the most pronounced in FMAs, followed by FPAs.

For inter-regional differences, the mean value of the Gini coefficient of the CDUFS was ranked mainly FPAs-FMAs (0.1538), FPAs-FBAs (0.1414), FMAs-FBAs (0.1157), so the difference of the coupling coordination between FPAs and FMAs were higher than those between other functional areas. The Gini coefficient of FPAs-FMAs showed a stable and continuous upward trend, from 0.0860 in 2000 to 0.1938 in 2019, with an obvious increase of 1.25 times, which further highlights the spatial non-equilibrium between FPAs and FMAs. The Gini coefficient of FPAs-FBAs had a relatively stable variation, increasing from 0.1210 in 2000 to 0.1429 in 2019, an overall increase of 18.10%. The Gini coefficient of FMAs-FBAs was the smallest in most years, with small differences between regions, but its overall growth trend is slightly fluctuating, increasing from 0.0970 to 0.1186 year-on-year, an increase of 22.27%. In contrast, the inter-regional difference of CDUFS between FPAs and FMAs showed a more prominent trend of expansion.

For the sources and contributions of regional difference, the mean contributions of intra-regional difference, inter-regional difference and transvariation density were 35.07%, 49.40% and 15.53%, respectively, showing that the inter-regional difference was the main source of overall difference in functional areas with a contribution of nearly 50%, followed by an intra-regional difference of functional areas and the smallest contribution of transvariation density. The contribution of inter-regional difference is in the range of 35.25% to 60.11%, basically showed a stable growth trend with an overall increase of 26.41%, except for a slight fluctuation before 2004. The contribution of intra-regional difference was in the range of 32.07% to 38.88%, showing a decline trend of first rising and then falling, but the volatility was relatively stable, with an overall decline of 9.46%. The contribution of transvariation density was in the range of 7.82% to 27.61%. Its changes were basically opposite to the inter-regional difference in a steady downward trend, with an overall decrease of 9.21 percentage points, indicating that the contribution of the cross-overlapping effect on the CDUFS between intra-regional and inter-regional to overall difference is gradually decreasing.

### 3.3. Dynamic Distribution

**Figure 5:**
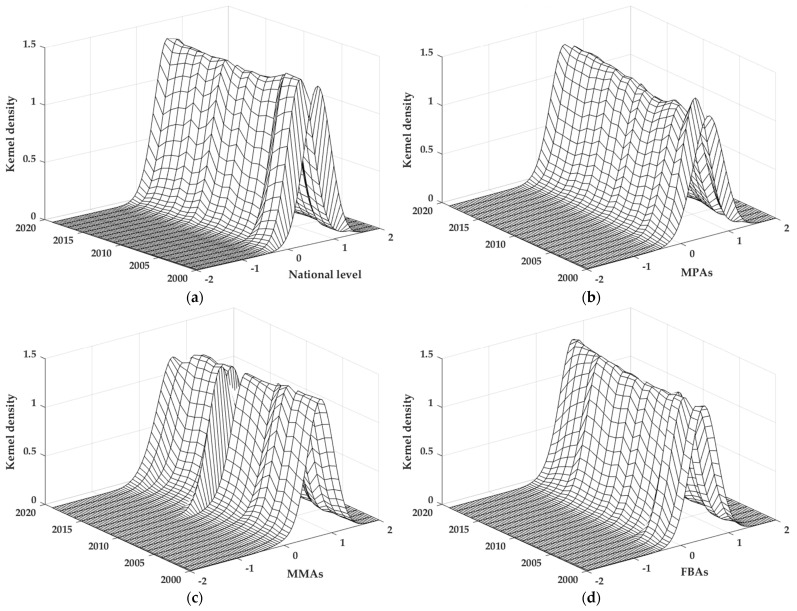
Kernel density estimation of the national level and different food functional areas. Note: This figure is drawn by the authors themselves using MATLAB based on the calculation results. (**a**) National level; (**b**) FPAs; (**c**) FMAs; (**d**) FBAs.

To further clarify the dynamic distribution of the CDUFS, the KDE method was used to portray the distribution location, distribution pattern, distribution ductility and polarization characteristics of the CDUFS at the national level and different food functional areas (Figure 5).

#### 3.3.1. The National Level

(1) Distribution location. The Kernel density curve at the national level showed an evolution process in which the curve center first shifted left and then right, and the peak height first increased and then decreased, indicating that the CDUFS generally showed a decreasing trend over time.
(2) Distribution pattern. The Kernel density curve showed a significant single-peak distribution with a relatively stable distribution trend. On the whole, the width of the main peak has increased, and the height of the main peak has decreased, indicating that the imbalance phenomenon in the CDUFS is still prominent, and the coordination gap between regions tends to expand, which is consistent with the typical characteristics in the previous section.
(3) Distribution ductility. The Kernel density curve had a weak left trailing phenomenon, and the distribution ductility was broadened to a certain extent but did not show a convergence trend. The gap of the CDUFS between the regions with high coupling coordination and the average level was somewhat enlarged.
(4) Polarization characteristics. The distribution of the Kernel density curve with a single-peak and a weak left trailing, indicating that the CDUFS did not show a polarization state and gradient effect, the single-peak distribution would continue to exist, and the gap of CDUFS between regions was more obvious.

The development of urbanization and food security have both made great progress to different degrees, but urbanization, which relies on economic growth, is growing faster, and food production still lags behind the urbanization process. In addition, there is heterogeneity in the endowment conditions and development environment among different regions. These are the realistic reasons for the single-peak distribution of CDUFS.

#### 3.3.2. Different Food Functional Areas

(1) Distribution location. The centers of Kernel density curve in both FPAs and FBAs evolved leftward and then rightward, with an overall shift to the left, similar to the national level, while the center of Kernel density curve in FMAs showed an obvious leftward evolution, indicating that the CDUFS in different food functional areas have a decreasing trend over time.
(2) Distribution pattern. The Kernel density curves of the three food functional areas were all significant single-peak patterns, with the width of the main peak increasing and the height of the main peak decreasing. Among them, the main peak height of FPAs showed a continuous decreasing process, while the main peak heights of FMAs and FBAs experienced a rising and then decreasing process, which generally indicates that the spatial imbalance of the CDUFS in different food functional areas is still prominent. The trends for this coordination gap widened.
(3) Distribution ductility. The Kernel density curve of FPAs did not show an obvious trailing phenomenon, while FMAs and FBAs showed left trailing phenomenon, and the FMAs were more prominent with a certain widening of the distribution ductility. The Kernel density curves of the three food functional areas did not show a convergence trend, and the gap between the areas with high coordination and the average level widened, prominently in the FMAs.
(4) Polarization characteristics. The Kernel density curves of different food functional areas with a single-peak indicates that the CDUFS of each food functional area did not have a polarized state and gradient effect, the single-peak distribution will continue to exist, and the coordination gap between regions is still obvious.

### 3.4. Evolution Trend

Although the Dagum–Gini coefficient and KDE can portray the regional differences and dynamic distribution of the CDUFS, it is difficult to deeply reflect the dynamic evolution trend of this spatiotemporal difference. So, the traditional and spatial Markov chain analysis were used to deeply examine the evolution trend of the CDUFS.

The basis of the Markov chain analysis is the transition probability matrix. It was necessary to divide the CDUFS of 330 prefecture-level and above cities during 2000–2019 into different types of state spaces. Based on the quantile division method [48], using the 1/4, 1/2 and 3/4 quantile as the boundaries, the CDUFS was divided into four adjacent but non-crossing completeness intervals: [0.0150, 0.2406], (0.2406, 0.2777], (0.2777, 0.3236], (0.3236, 0.5663], the completeness intervals of these four state types can be represented by *k* = 1, 2, 3, and 4, respectively. The larger *k* is, the higher CDUFS is (lower, low, high, higher).

#### 3.4.1. Traditional Markov Chain Analysis

The traditional Markov transition probability matrix was calculated based on the division of state types (Table 4). The elements on the diagonal line represent the probability that the state type of the CDUFS does not transfer in a region, which reflects the stability of the evolution trend of the CDUFS in this region, while the elements on the non-diagonal line represent the probability that the CDUFS in a region transfers between different state types. Therefore, without considering the spatial effect, the evolution of the CDUFS is characterized as follows:

(1) The CDUFS had the stability of keeping the original state. All the elements on the diagonal were larger than those on the non-diagonal significantly, implying that the CDUFS had a smaller probability of state transfer. The fluidity among states was low with an obvious path-dependence. In addition, the probability of maintaining stability was greatest for the types at either end of the diagonal (types 1 and 4), while types 2 and 3 in the diagonal had a relatively smaller probability of maintaining stability. The CDUFS was more likely to be distributed at lower and higher levels, low and high distributions more prone to transfer.
(2) Areas with a higher level of CDUFS were more likely to transfer to the lower level, while areas with a lower level of CDUFS had a higher likelihood of shifting to a higher level. For example, the probability of type 4 transition downward was 0.1091, the probability of type 3 transition downward was 0.1073, the probability of type 2 transition downward was 0.1013, 0.1091 > 0.1073 > 0.1013. It can be seen that areas in type 4 were more likely to transfer downward. Although the evolution trend was stable in areas with a high level of CDUFS, there is a certain risk and possibility of falling back, which requires vigilance and attention. In addition, the probability of type 2 transferring upward was greater than that of type 3 (0.1742 > 0.1171), indicating that areas with a relatively low CDUFS have higher room for growth.
(3) The CDUFS was difficult to achieve a leapfrog evolution in the short term. The probability transition of the CDUFS occurred almost on both sides of the diagonal. For elements on both sides of the non-diagonal, the probability values were significantly smaller than those on both sides of the diagonal. For example, the probability of type 2 transferring upward to type 4 was 0.0083, which is obviously smaller than the probability of transferring to type 3 (0.1389). Among two consecutive years, the probability of achieving a leapfrog transfer was low (e.g., 1→3, 1→4), which means that the evolution of CDUFS in each region was a relatively stable and continuous process.

#### 3.4.2. Spatial Markov Chain Analysis

The probability transfer matrix of the traditional Markov chain did not consider the spillover effect of type of transfer in neighboring regions, but the CDUFS had a significant positive correlation and dependence in space. Therefore, the type of transfer of the CDUFS is not isolated in space but is complementary and effectively linked to the surrounding areas. Introducing the spatial lag effect, the spatial Markov chain transition probability matrix (Table 5) was constructed based on the spatial lag type of each area in the initial year. In addition to the common features with the traditional Markov chain transition probability matrix, it also had the following spatial evolution features.

**Figure 6:**
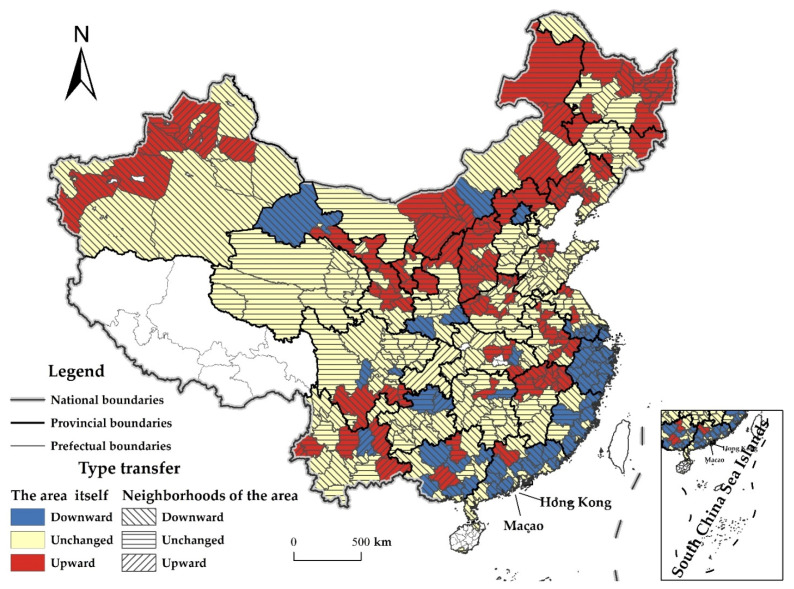
Spatial distribution of CDUFS type of transfer under the spatial Markov chain. Note: This figure is drawn by the authors themselves using ArcGIS based on the calculation results.

(1) Geospatial pattern plays an important role in the dynamic evolution of the CDUFS, and spatial Markov chain analysis can provide a spatially meaningful interpretation of the single-peak distribution pattern of the CDUFS. Under the spatial effect, the type of transition probability of the CDUFS in each area was not the same, was also not equal to the corresponding traditional Markov transition probability matrix, otherwise, the effect of spatial lag would not exist (Figure 6). For example, when the spatial effect was not considered, the transition probability from type 2 to type 3 in an area was P_23_ = 0.1389. When the area was adjacent to an area in type 2, P_23__|__2_ = 0.1019, when it was adjacent to an area in type 3, P_23__|__3_ = 0.1529, and when it was adjacent to an area in type 4, P_23__|__4_ = 0.2237. It can be seen that it is necessary to consider the spatial background when analyzing the spatial evolution of the CDUFS. An area is adjacent to the areas of different types, the state transition probability of the CDUFS in this area will be different. In general, for a certain area, the probability of upward transfer of its type was greater when adjacent to areas with higher coordination, while the probability of downward transfer of its type was greater when adjacent to an area with lower coordination.
(2) The evolution of the CDUFS remained more stable in its original state; the possibility of jump transfer is low. This is because the elements on the diagonal were still larger than the elements on the non-diagonal after considering the spatial background. As the spatial lag type rises, there are differences in the stability and transition probability of the evolution of the CDUFS. For areas with a lower CDUFS (type 1), their stability decreased as the spatial lag type increased, but the probability of upward transfer was increasing. For areas with a low CDUFS (type 2), their stability showed an increase and then decrease with the increase of spatial lag type, while the probability of upward transfer showed a decrease and then increase, and the probability of downward transfer in decreasing. For areas with a high CDUFS (type 3), their stability also showed an increase and then a decrease with the increase of spatial lag type, but the probability of upward transfer was increasing and the probability of downward transfer was decreasing. For areas with a higher CDUFS (type 4), their stability decreased and then increased with the increase of the spatial lag type, while the probability of downward transfer showed an increase and then a decrease.

## 4. Discussion

While previous studies have focused more on the one-way impact of urbanization on food security and the challenges faced by food security in the urbanization process [51], this paper innovatively explores the interaction between urbanization and food security. The CDUFS always fluctuates in the low coordination interval, which is different from the study of Yao [20]. Both urbanization and food security have achieved stable growth, but the interaction effect between the two has not achieved synchronous growth. It mainly stems from the fact that the growth of food security continues to lag behind urbanization development, and the unbalanced development of the two constrains the improvement of coupling coordination level. In addition, the distribution pattern of the agglomeration of the CDUFS basically keeps a steady trend and shows a “center-periphery” with FPAs in the north gradually decreasing to FBAs in the northwest and FMAs in the south.

The CDUFS of China show obvious regional differentiation, and the overall regional differences of the CDUFS in China have expanded. The possible explanation is that, although food security is increasingly guaranteed, the growth process of urbanization is still faster than the supply capacity of food production. It has been found that urbanization inevitably brings about the reallocation between urban and rural areas [22], and the regional differences in endowment conditions and industrial structure also make the relationship between urbanization and agricultural production exist in regions located in different geographical spaces. Continued urbanization and changing consumption patterns pose challenges to food security. Avoiding deterioration in food security depends on the responsiveness and resilience of the smallholder farming sector. Rural-urban food supply linkages [52] and policy synergies are also critical.

For regional differences in different food functional areas, inter-regional differences are the main source of overall differences, mainly in the widening trend of differences in coupling coordination between FPAs and FMAs, and the internal differences of FMAs are also expanding. The FPAs face the dual pressure of ensuring food security and urbanization development, and the supply task of food production in FPAs is equally important in the urbanization process, while FMAs basically cover the more economically developed areas of East China and the South China coast. The industrial structure upgrading implies the proportion of agriculture is declining. There is the adjustment of structure in food production to make room for urbanization development, and the coordination level between urbanization and food security is poor.

The regional differences between FPAs and FMAs make us alert to a mismatch between the main body of food production and the spatial distribution of soil-water resources in the South and North [53]. The production and marketing pattern of “northern food transportation to the south” remains stable. The main producing areas undertake more food supply tasks, but their soil-water conditions do not have resource advantages, and the gap between grain supply and demand is gradually increasing in some northern provinces of main producing areas. Therefore, it is possible to explore the pilot mechanism of interest linkage between the main producing areas and the main marketing areas. Through the pilot project, according to their own development base and the total amount of grain transferred, the main marketing areas in the southern area can compensate for the benefits of the main producing areas in the northern area, promote agricultural investment in the main producing areas in order to guarantee grain production in the main producing areas, and maintain a long-term stable inter-regional relationship between grain supply and demand, so that the main marketing areas can also assume a certain responsibility for ensuring food security in the form of trade.

Amongst the several challenges, food security will be a serious issue for the future of high-quality urbanization [54]. This study is of great significance for understanding the coordinated relationship between urbanization and food security, exploring a sustainable path for high-quality urbanization and guaranteeing food security in China, and promoting a synergistic balance between urbanization and agricultural production.

(1) Changes in spatial use of territory land led to the conflict between urbanization and food security. It is necessary to plan the urban-rural land use scientifically and reasonably, clarify the limit of urban land, and slow down the expansion speed of urban land. In addition, we should strengthen the policy of farmland protection in the urbanization process, strictly guard the red line of cropland use, and optimize the balance management system of farmland occupation and supplement to maintain the dynamic balance of total farmland, so as to guarantee the high-quality development of urbanization and sustainable food production.
(2) Urbanization not only leads to the adjustment in production resources and grain-growing behavior, but also provides an opportunity for large-scale, mechanization and efficient operation of food production [55]. Therefore, the process of high-quality urbanization involves guiding households to transfer management rights of land orderly to bring into play the scale effect of land. Improving food production efficiency and yield capacity through intensive land use and technological progress can also provide useful support for urbanization, which promote the coordinated development between urbanization and food security.
(3) Differences in industrial division of labor make regional differences in the CDUFS, and there is no convergence in the long-term evolution process. Therefore, the strategies of urbanization development and ensuring food security by different regions should be adapted to local conditions, and in line with their own development positioning based on industrial characteristics.
(4) The FPAs need to grasp the room and direction in the process of urbanization, avoid the irregular expansion of urban space, ensure the free mobility of factors between urban areas and rural areas, adapt the utilization structure of water-soil resources, enhance the efficiency of land use, and drive the intensive management of food productive through urbanization. The FMAs have a high level of urbanization, and with the industrial structure upgrading, the comparative advantage of agricultural production has declined obviously. It should focus on weighing the intrinsic structure of food production against the extrinsic changes triggered by urbanization and improve the factor agglomeration and labor productivity through technological progress. The urbanization of FBAs is relatively backward, but grain producing and marketing basically maintain a balance. Therefore, the potential for coordination between urbanization and food security is greater. The trade-off between the government’s guiding effect and the market’s allocation effect should be focused on to optimize the regional layout of urbanization and food production by effective cooperation between government and market.

Based on the panel data of prefecture-level, this study allowed for a more detailed exploration on a smaller dimension. However, due to the difficulty of obtaining data on relevant indicators, food security is represented only as a single indicator. More complete data acquisition is what needs to be improved in subsequent research.

## 5. Conclusions

This paper measures the CDUFS in China using prefecture-level city panel data and the coupled coordination degree model based on understanding of the spatial correlation and differentiation of the CDUFS by ESDA. From the perspective of different food functional areas, regional differences, and sources, distribution dynamics and evolutionary trends of the CDUFS were analyzed in depth using the Dagum–Gini coefficient, the Kernel density estimation and the spatial Markov chain. The main findings are as follows:

(1) The CDUFS in China showed a downward trend in fluctuating within the low coordination range, and food security continues to lag behind urbanization. The regional differences of the CDUFS are obvious with a continuously enhanced positive correlation in space. This exhibits a stable distribution pattern of H-H in the Northeast Plain and Huang-Huaihai Plain, and L-L in the Northwest, Southwest and Southeast coast.
(2) There were obvious regional differences in the CDUFS in China, and the overall differences have expanded. For different food functional areas, inter-regional differences were the main source of contribution to the overall differences, with the highest regional differences between FPAs and FMAs, and their spatial imbalance gradually prominent. The contribution of intra-regional differences was the second, with the highest of internal differences in FBAs, and its expanding trend of internal differences was more obvious. The contribution of transvariation density was the smallest.
(3) The main peak of the distribution curve of the CDUFS in China has increased in width and decreased in height. The imbalance of the CDUFS between regions is still prominent, but it does not show a convergence trend, and the single-peak distribution will continue to exist. The CDUFS in the three food function areas show a significant leftward shift of the single-peak distribution process, and the distribution curve of the FPAs do not show a trailing phenomenon. While the FMAs and FBAs show the left trailing, the FMAs are more obvious.
(4) The CDUFS in China have the stability of maintaining the original state, obviously path dependent. The trend of the CDUFS transferring to a high level is not obvious, but areas with higher CDUFS have a higher possibility of transferring to a low level. It is difficult to achieve a leapfrog evolution of the in the short term. Geospatial pattern plays an important role in the dynamic evolution of CDUFS, and there are differences in the stability and transfer probability of the CDUFS evolution under the different spatial lag type. The long-term evolution trend of CDUFS in China is influenced by the geospatial effect, but the single-peak pattern is relatively stable and does not exhibit significant convergence in the long term.

## Figures and Tables

**Table 1:** Brief descriptive statistics.

Variables			Variable Desceition	Mean	Std.
CDUFS	Urbanization	Population urbanization	urbanization rate of resident population	0.2824	0.0631
		Land urbanization	urban built-up area/land area		
		Economy urbanization	non-agricultural industries/GDP		
	Food security	Food output per capita	food production/total population		

**Table 2:** Moran’s I of the CDUFS.

Year	Moran’s I	Z	Year	Moran’s I	Z	Year	Moran’s I	Z	Year	Moran’s I	Z
2000	0.473	13.615	2005	0.623	17.904	2010	0.655	18.812	2015	0.652	18.734
2001	0.353	10.189	2006	0.617	17.74	2011	0.587	16.894	2016	0.654	18.782
2002	0.483	13.914	2007	0.603	17.337	2012	0.669	19.223	2017	0.667	19.156
2003	0.452	13.044	2008	0.637	18.292	2013	0.665	19.099	2018	0.674	19.358
2004	0.607	17.449	2009	0.621	17.847	2014	0.642	18.452	2019	0.649	18.670

**Table 3:** Regional differences and their sources of CDUFS in different functional areas.

Food Functional Areas		2000	2003	2006	2009	2012	2015	2018	2019	Mean
Intra-regional difference	FPAs	0.0742	0.0848	0.0950	0.0983	0.1008	0.0965	0.0948	0.0882	0.0933
	FMAs	0.0750	0.0812	0.0838	0.0956	0.0968	0.0992	0.1116	0.1126	0.0958
	FBAs	0.0959	0.1255	0.1217	0.1352	0.1261	0.1133	0.1185	0.1003	0.1181
Inter-regional differences	FPAs-FMAs	0.0860	0.1000	0.1254	0.1602	0.1772	0.1814	0.2065	0.1938	0.1538
	FPAs-FBAs	0.1210	0.1293	0.1449	0.1540	0.1475	0.1420	0.1481	0.1429	0.1414
	FMAs-FBAs	0.0970	0.1087	0.1060	0.1200	0.1211	0.1177	0.1334	0.1186	0.1157
Contribution rate/%	Intra-regional	35.42	37.15	36.35	35.18	35.26	34.44	32.92	32.07	35.07
	Intra-regional	47.55	35.25	46.16	48.22	51.14	53.78	57.11	60.11	49.40
	Transvariation density	17.03	27.61	17.49	16.60	13.60	11.78	9.97	7.82	15.53

**Table 4:** Traditional Markov chain transition probability matrix for CDUFS.

Type	n	1	2	3	4
1	1614	0.8482	0.1363	0.0105	0.0050
2	1570	0.1013	0.7516	0.1389	0.0083
3	1537	0.0078	0.0995	0.7755	0.1171
4	1549	0.0039	0.0077	0.0975	0.8909

**Table 5:** Spatial Chain Transition Probability Matrix for CDUFS.

Spatial Lag	Type	n	1	2	3	4
1	1	250	0.9120	0.0840	0.0040	0
	2	116	0.1121	0.7586	0.1293	0
	3	41	0.0244	0.2683	0.6829	0.0244
	4	11	0	0	0.0909	0.9091
2	1	736	0.8641	0.1209	0.0095	0.0054
	2	589	0.1121	0.7827	0.1019	0.0034
	3	233	0.0258	0.1760	0.7425	0.0558
	4	95	0.0211	0.0211	0.1263	0.8316
3	1	595	0.8151	0.1681	0.0118	0.0050
	2	713	0.1010	0.7377	0.1529	0.0084
	3	833	0.0060	0.0960	0.8019	0.0960
	4	595	0.0050	0.0118	0.1193	0.8639
4	1	33	0.6061	0.3030	0.0606	0.0303
	2	152	0.0526	0.6908	0.2237	0.0329
	3	430	0	0.0488	0.7512	0.2000
	4	848	0.0012	0.0035	0.0790	0.9163

## Data Availability

The data presented in this study are available on request from the corresponding author. The data are not publicly available due to data management.

## Abbreviations

CDUFS	coupled coordination degree between urbanization and food security
FPAs	food main producing areas
FMAs	food main marketing areas
FBAs	food balanced areas
